# Medical Nature-Based Rehabilitation Program for Individuals with Exhaustion Syndrome: Changes in Quality of Life, Exhaustion Symptoms and Overall Health

**DOI:** 10.3390/ijerph20176677

**Published:** 2023-08-29

**Authors:** Eleanor Petitt, Bo Rolander, Per Johnsson

**Affiliations:** 1 Department of Psychology, Lund University, 221 00 Lund, Sweden; 2 Futurum—The Academy for Health and Care, Region Jönköping, 553 05 Jönköping, Sweden

**Keywords:** nature-based rehabilitation, nature-assisted interventions, stress, Exhaustion Syndrome

## Abstract

Stress-related health problems have increased sharply over the last two decades and have become a serious issue at all levels of society. In the Jönköping Region in southern Sweden, a nature-based rehabilitation (NBR) program for adults with Exhaustion Syndrome has been developed and then implemented into the Swedish National Healthcare System. The main aim of this study was to investigate the effectiveness of this NBR-program. This was achieved by examining patients’ quality of life, exhaustion symptoms and overall health using self-assessment instruments, comparing the results before participation to immediately after, three months after and six months after. With a sample size of 67 participants, the results show a statistically significant improvement for all points. From a public health perspective, and with background knowledge of the nature of the patient group under treatment, the studied program would appear to be effective and economic, having a satisfied patient group as well as a favourable comparison with the outcomes of other research programs. Although the results are promising, as this is a naturalistic field study, there is no control group, and further research is encouraged. We suggest randomised controlled studies, longitudinal studies and investigation of mediators.

## 1. Introduction

### 1.1. Stress-Related Health Issues

The concept of stress was introduced into main-stream medical thinking in the 1930s by Hans Selye and is defined as an individual’s reaction to excessive strains or overloading [1]. There is a broad consensus concerning the definitions of stress: it is perceived as the difference between the challenges and demands made on the individual (whether by herself and/or the environment) and her actual ability to manage them. From this perspective, stress can be described as a condition of heightened psychological and physiological preparedness, a state similar to that in which the individual anticipates that some form of danger or challenge may be approaching and to which she responds by preparing for fight or flight. This preparation involves the sympathetic nervous system being activated and physiological resources being reorganised to be more effective in meeting possible threats. For example, blood flow is directed from the digestive system to the larger muscles, heart- and breathing-rates increase in order to provide the muscles with more oxygen and production of the stress hormones adrenalin and noradrenalin is raised. Stress responses such as these are not dangerous when they exist for short periods of time, but they can be physically, cognitively and emotionally debilitating if they persist over a long time [1,2,3,4,5,6]. Chronic exposure to stress means that the body is also exposed to cortisol for a prolonged time, which can possibly lead to secondary problems such as overweight, cardiovascular diseases, higher risk for infections, metabolic syndrome and mental health issues [1,7,8].

Stress-related health issues are categorised as psychosomatic and have become increasingly more common [8,9,10]. Since 2005, “Exhaustion Syndrome”, synonymously named Exhaustion Disorder in the literature, has been included in ICD-10 SE, the illness and disease classification manual used by the Swedish National Health Service [11]. The term was formally recognised as a medical diagnosis by the Swedish National Board of Health and Welfare in 2010 [12]. 

Stress-related health problems affect individuals who have been exposed continuously to high levels of stress over a long time without having the possibility to recover and recuperate. This, in turn, can lead to the onset of Exhaustion Syndrome, which is a serious and long-lasting state. Exhaustion Syndrome is a fatigue-dominated diagnosis [13], which is characterised by both a severe physical and a cognitive tiredness which do not cease, even when the individual is able to rest and sleep. Other typical physical symptoms include physical tiredness and exhaustion, disruption of sleeping patterns, dizziness, heightened risk of infection and changed hormonal levels—for example, of cortisol [12]. Cognitive symptoms include disturbances to both working memory and short- and long-term memories, weakening of executive functions, decrease in attention, difficulties in concentration, lack of mental stamina, low stress tolerance, oversensitivity to sound and light and extreme mental tiredness. Other common symptoms are lack of psychological energy that results in a weakened ability to carry out tasks; a much longer recovery time after being subjected to psychological or emotional stress; extreme mental tiredness when subjected to stress and difficulty in relaxing even when extremely tired [4,12,14,15]. Qualitative studies have found that individuals diagnosed with Exhaustion Syndrome experience existential struggles and that their lives are heavily impacted on a mental, cognitive, physical and social level [16,17]. This was found both in the early stages of the illness as well as a decade later [16,17]. Exhaustion Syndrome also often generates negative emotions and even secondary depression [18,19].

Beginning in the 1990s, in Sweden, there has been a significant increase in long-term sick-leave that has a psychological basis [20], and, by 2010, diagnoses that identify poor mental health constituted the fastest-growing group of explanations as to why individuals were granted sick-leave [15]. The Swedish Social Insurance Agency [9] found, in 2020, that Exhaustion Syndrome had become the number one cause for long-term sick leave for psychiatric and somatic illnesses in Sweden, and a recent report from the Swedish Department of Social Insurance states that anxiety and stress-related illnesses experienced by women make up 48% of the sick-leave granted [21]. A large part of this can be linked to work-related stress [22]. Sick-leave influences not only the individual’s financial situation, but also subsequent loss of productivity, and healthcare and medical costs affect the national economy [23,24].

Stress-related health issues are, thus, not only a burden for more and more individuals, but have also become a significant socio-economic factor as well as an increasing strain on the healthcare system. Initially, research tended to focus on why people developed stress-related symptoms, but, since 2000, more time has also been spent on examining the consequences of contracting such problems [15,25,26]. Research has also developed a greater interest in the treatment of stress related health issues such as Exhaustion Syndrome [26,27,28,29,30,31,32,33,34,35,36,37,38,39]. One group of treatments that has attracted increasing attention is nature-supported treatment interventions, where nature is used to promote better health [28,40,41,42].

### 1.2. Nature-Based Rehabilitation

There exists a long tradition of seeking both the assistance of nature to support health development [43] as well as the potential therapeutic value to be found in developing a relationship with animals [44]. The systematic use of human–animal interaction for therapeutic purposes can be found as early as the mediaeval period [45], and even Hippocrates used his garden to assist in promoting the experience of well-being [46]. At the present time, there are a number of different interventions which use nature, sometimes including animals, in the promotion of health [47,48,49,50,51]. Some examples are horticulture therapy, where participants engage in plant-based activities and gardening following a treatment plan and facilitated by a horticulture therapist [29]; forest bathing, an old tradition originally from Japan where immersion in a forest setting is used to promote health [36]; traditional care and treatment in habilitation or physical rehabilitation taking place in an outdoor environment [47,52]; NBR with therapeutic use of farming activities and agricultural landscapes, facilitated by the farmers and weekly follow-up with healthcare personnel [47] and nature-based rehabilitation where a multi-disciplinary team facilitate participation according to a treatment plan [53]. The term Medical Nature-Based Rehabilitation (MNBR) has not yet started to emerge in the literature, but is used when referring to NBR where licensed healthcare personnel are involved. 

Studies, both quantitative and qualitative, have found that NBR can be helpful for decreasing symptoms of exhaustion, severe stress, depression and anxiety, increasing general psychological wellbeing and overall health, having a positive impact on restarting stalled rehabilitation processes and functioning in everyday life as well as increasing likelihood to return to work [27,28,31,41,51,54]. In Sweden, NBR has been a research subject since the turn of this century [28,30,31,32,42,47,55], and a great deal of research has focused on stress-related health issues, often revealing positive results [30,31,40,46,56,57]. However, collaborative research from different health institutes in Sweden has concluded that the effectivity of such programs is still uncertain and needs more quantitative studies [58]. NRB for stress-related health issues varies both in their length, the number of hours, and the composition of the professional team differs [31,40,47,59]. 

Common to the theories and hypotheses that underlie NRB is the assumption that being present, using all of our senses, in nature and interacting with animals can be of help in the promotion of health [29,40,60,61,62]. Empirical studies of programs that use such interventions appear to support this assumption [28,30,56,62,63]. *The Attention Restoration Theory (ART*) is relevant when it comes to cognitive recovery from stress and focuses on two types of human attention [64,65]. *Directed attention* helps us to stay focused on a task, as “unnecessary” stimuli in the environment are disregarded and intense concentration is used. *Soft fascination* describes a form of effortless, spontaneous attention not controlled by will, but rather lands on things in the surroundings that do not demand our effort. Directed attention is seen as a limited resource that needs to be recharged, which is promoted in restorative environments—such as nature—where soft fascination occurs unconsciously [40,66]. *The Psycho-Evolutionary Theory* states that humans have adapted to surviving in nature and, therefore, have positive reactions in such an environment [40,67,68]. Non-threatening nature settings are thought to promote a positive mood and quicker physiological recovery (such as lowered blood pressure and muscle activity) from acute stress than in urban environments [40,67]. *The Savanna Hypothesis* argues that habitat preferences such as open landscape and closeness to water were formed during evolution as these facilitated survival, which is why these features are appealing to us still today [68]. *The Prospect Refuge Theory* describes the optimal natural environment to be a protected area with a view. Such a place would have been advantageous in human evolution by providing both shelter with a possibility to hide and a lookout point to identify different kinds of danger or opportunities [69]. “Biophilia” is defined as “the urge to affiliate with other forms of life” and is the foundation of *The Biophilia Hypothesis*, that states that humans have an innate ability and tendency to connect with nature and its inhabitants which has helped human survival [70]. A theory that presents different needs for supportive physical and social environments, depending on a person’s physical and mental capacity and executive functioning, is the *Theory of Supportive Environments (SET)*, that describes a connection between how the individual is feeling and what her needs are for the surrounding physical and social environment, especially in different stages of stress-related health issues [46,71]. The positive impact that nature and animals can have on people has also been found in biological markers such as lowered cortisol and rise in oxytocin [72,73,74,75]. Evidence that spending time in forests and gardens positively impacts the immune system and decreases activity in brain regions associated with stress has also been presented [47,76,77]. 

### 1.3. The Studied Medical Nature-Based Rehabilitation Program 

Patients between the ages of 18 and 65 were referred to the program by their doctor from local healthcare centres, psychiatric outpatient units or occupational healthcare units. Referred patients were then invited for an assessment interview with a psychologist and an occupational therapist. The interview had two major aims: firstly, to inform the potential participant about the program to see if she was interested and, secondly, for the clinicians to assess if the patient and the program seemed to suit each other. The primary criterion to be admitted to the program was that the individual’s main problem was the presence of stress-related mental health issues (not including traumatic stress as a primary diagnosis), even if accompanied by secondary or simultaneous illnesses such as depression, anxiety or long-term pain. The level of illness had to be assessed to match the level of intensity in the program.

The professional team consisted of a gardener (who was also the project leader), a licenced psychologist and a licensed occupational therapist, all of whom were knowledgeable about nature-based intervention and rehabilitation. The occupational therapist’s dog was also considered a member of the team and joined the group at the start of each day, during the breaks and at other times deemed suitable by the occupational therapist. As the team included licenced healthcare professionals, the program was classified as an MNBR-program, but is often referred to herein as simply NBR.

The facility consisted of a house and a garden located beside a nature reserve. The house had the space needed for group sessions, a large work bench for nature-assisted activities, a room where the participants could retreat if they so wished, a kitchen and rooms for the staff.

The nature incorporated in the program was chosen and designed with previous research in mind. The garden satisfied the suggestions offered by Grahn and Ottosson [46], containing both open and more secluded spaces, water, a fireplace, flower beds, herb beds, vegetable plots and fruit trees. The nature reserve offered various kinds of nature, including different kinds of forest, open spaces, water and hills. This was in accordance with the Savannah Theory (68), the Theory of Supportive Environments (46,71) and the Prospect Refuge Theory (69). Many opportunities that enabled soft fascination were presented (64,65). Decisions concerning activities had a degree of flexibility, allowing them to accommodate to the actual needs of the group. A neighbouring agricultural gymnasium possessed a number of horses and calves, and these could be visited by prior agreement when there were no other activities. 

The NBR-program was designed to help adults with stress-related mental health issues by providing a structured group intervention that aimed to improve their health in two major ways: through improving their ability to cope with the demands of their daily lives and through the experience generated by the consequences of their impaired resources. The program goals were to improve general health and quality of life for the patients; to reduce exhaustion symptoms; to improve mental and emotional health and to promote the ability to cope with the demands of everyday life. 

Group size varied between six and eight patients and the program was ten weeks in length. The participants came to the facility twice per week for three hours each time. These three hours were typically divided into two treatment modules, with a tea and coffee break in between. Three months after finishing the program, the group was invited back for a reunion around the fireplace in the facility’s garden. This can be thought of as a booster session that offered the patients an opportunity to meet and reflect upon their progress. 

The NBR-program was started in 2012 within the Swedish Healthcare System of the Jönköping Region in southern Sweden. As a consequence of staff changes in 2015, new structure and content were designed (described below). One of the changes was the introduction of self-assessment evaluation scales. These changes marked the starting point of this study’s timeframe. 

The programs’ psychological treatment was based on Cognitive Behavioural Therapy (CBT), which integrates cognitive psychotherapy and behavioural therapy based on the conception that we can increase our well-being by changing our thought and behaviour patterns [78]; Acceptance and Commitment Therapy (ACT), often referred to as the third wave of CBT, rests on the theoretical framework of Relational Frame Theory [38], emphasising the importance of acceptance, mindfulness and value-based skills to obtain psychological flexibility [79]. Mindfulness, an approach to life that originated from Buddhist psychology and was later adopted by Western psychology, focuses on being present, aware and accepting in each moment [80,81]; this was incorporated into all parts of the program.

During the program, a group meets twice per week. Typically, Day 1 begins with a semi-structured group treatment followed by a walk in the surrounding nature, that includes a time for stillness and silence. Day 2 normally includes a nature-based activity and a walk in the surrounding nature, again including time for stillness in silence. During the coffee break between the two sets of activities, the patients can choose to socialise with the other group members, the leaders or the dog, or to find their own space and sit by themselves. 

The group treatment consists of eight semi-structured sessions led by the psychologist (sometimes together with the occupational therapist). The very first day is designed as an introduction to the program and the time is used by the occupational therapist to introduce theories underlying occupational therapy. Self-assessment scales, described below, are filled in on this first day. The occupational therapist and the psychologist share responsibility for Day 1 and both are always present for the activities that follow the group treatment sessions. 

The nature-based activities typically consist of gardening, activities in the forest, animal-assisted activities led by the gardener or nature-assisted occupational therapy led by the occupational therapist, which will often reflect the different seasons. Some examples are picking apples, making apple juice, carving pumpkins, making Christmas decorations out of pine branches, planting seeds or cuttings, digging in flower beds or vegetable plots, wood carving, hiking and gathering around a fire, dry-felting and meeting horses, calves or guinea pigs. Depending on the group’s needs, the occupational therapist may use one of these days or parts thereof to discuss theoretical aspects of occupational theory with the group. The occupational therapist and the gardener together have responsibility for Day 2 and are both always with the group, while the psychologist is often on hand and ready to assist. 

The aspect of nature chosen each time for activities and walks was decided using Grahn’s pyramid, coupled with the information that the team gathered from the participants concerning their current state [46,71]. For a typical program schedule, please see Appendix A.

At the end of the rehabilitation program, each patient, the occupational therapist and/or the psychologist met with the patient’s referring doctor and other relevant agents such as their employer, Social Insurance Agency Officer or Employment Agency Officer to participate in discussions concerning possible rehabilitation plans. The self-assessment scales were filled in again by the patients on the last day of the program, together with an evaluation form that included a patient satisfaction Likert scale. At the booster session, the same scales were once again filled in, and a six-month follow-up was sent to the patients via mail.

## 2. Aims and Objective

The main aim of this study is to investigate the effect of a specific NBR-program for adults with Exhaustion Syndrome. This was achieved by investigating patients’ quality of life, exhaustion symptoms and overall health using self-assessment instruments, comparing the results before participation to immediately after, three months after and six months after. A further aim is to find out to what extent the patients were satisfied with the program. To determine this, results from a client satisfaction survey were studied. 

## 3. Materials and Methods

### 3.1. Participants

The participant were patients that attended the NRB-program as part as their healthcare for Exhaustion Syndrome. The age span was between 27 and 63 years, with a mean age of 43.9 years. In all, 68% (SD = 9.7) of the participants were between 33 and 53 years of age; 91% of the participants identified as female and 9% as male. To be included in the study, the patient had to have attended at least half of the program/scheduled sessions and filled in the self-assessment scales at the start and the end of the program.

On the whole, 113 patients attended the first 16 groups, which were chosen to be included in the study. Of these, 93 had attended at least half of the scheduled sessions and completed the self-assessment scales both at the beginning and at the end of the program and were, therefore, considered eligible for the study. The 93 patients were given written information about the study as well as a consent form and were asked if they wanted to participate. Of the eligible participants who were asked for their informed consent, 5 declined and 21 did not reply; 67 of the patients gave their informed consent and are included in this study. Further to this, 61 participants had completed at least one of the self- assessment scales at the three-month follow-up and at 44 had completed at least one of the self-assessment scales at the six-month follow-up.

### 3.2. Instruments

When the NRB-program was first started, a decision was made to include validated self-assessment scales as part of the clinical routine in order to continuously monitor patient health, to obtain feedback on the clinical work and to help to evaluate and improve the program. The first group was used as a pilot project. The scales were distributed four times: on the first and last days of the rehabilitation program and three and six months after completion of the program. Participants were asked to complete the evaluation form on the last day of the program. The scales chosen were EQ-VAS [82], Brunnsviken Brief Quality of Life Scale (BBQ) [83] and Shirom–Melamed Burnout Questionnaire (SMBQ) [84]. 

*Shirom–Melamed Burnout Questionnaire (SMBQ)* is a quantitative self-rating scale that is designed to explore the incidence and degree of a burnout syndrome. It consists of 22 items, divided into 4 sub-scales: Physical Fatigue (8 items); Cognitive weariness (6 items); Tension (4 items) and Listlessness (4 items). Each item is rated using a seven-point scale ranging from 1 (“Never or almost never”) to 7 (“Always or almost always”). Five of the items have a reversed scoring (one in the Tension domain, three in the Listlessness domain and one in the Physical Fatigue domain). For each sub-domain, the average is calculated by dividing the score by the number of items. The average for all 22 items is called the SMBQ-Global. The cut-off point for SMBQ-Global is 3.75, where high (pathological) burnout is indicated by a score ≥ 4.47 and low (healthy) burnout by a score of ≤2.75. The cut-off point for the non-clinical population is 3.75; Cronbach’s alfa: a = 0.91 [84]. The validity of the SMBQ instrument has been tested in research [85] and it was concluded that the measure could be used in both clinical work and in research to determine the level of exhaustion. They also concluded that the domain “Tension” contained weaknesses and proposed a revised version from which that domain was excluded. Our decision to adhere to the first version of SMBQ in the present project was motivated by the experienced value of the domain “Tension” in clinical work [85]. SMBQ has a good reliability (Cronbach’s alfa of 0.94) [86]. SMBQ has been used extensively both in Sweden and internationally [87,88,89].

*Brunnsviken Brief Quality of Life Scale (BBQ)* is a self-rating scale designed to quantify the individual’s subjective experience of her life-quality. It was developed from the more extensive self-rating questionnaire *“Quality of Life (QOLI)”* [90], to make its application in everyday clinical practice easier by reducing the number of questions. The instrument consists of 12 paired statements that address 6 areas of life in terms of satisfaction and importance. Each item is rated by the respondent using a five-step Likert scale, where 0 signifies “Strongly disagree” and 4 signifies “Strongly agree”. The BBQ total score is reached by multiplying the points received within each pair with each other (Importance × Satisfaction) and then adding the six results together (minimum 0 points, maximum 96 points). BBQ is sufficiently sensitive to distinguish between non-clinical and clinical groups [91]. Maximised classification accuracy (cut-off score) is reached at a score of 52.5, with a specificity of 0.71 and a sensitivity of 0.79. BBQ has been adapted to Swedish norms, with a relatively good internal consistency (Cronbach’s alpha of 0.68) and a good test–retest reliability (test–retest coefficient of 0.89). The validity of BBQ has been proven to be as good as that of QOLI which is considered good [91].

*EQ-VAS* is a self-rating scale where the respondent judges the quality of her present health by making a cross on a vertical scale that starts at the bottom with 0 (worst possible health) and goes up to 100 at the top (best possible health) [83]. In this way, EQ-VAS generates an individually rated measure of health that can be used in a quantitative way [83]. Studies have shown that EQ-VAS is a standardised instrument that is of practical value and possesses both good reliability and validity [92].

In addition, the patients were asked to fill in an evaluation form, created within the program, that asked questions regarding how they had experienced the program, what they thought was of value and not of value, what they thought could have been different and what they appreciated. A Likert scale regarding client satisfaction with the program was added to the evaluation and the four choices offered were “completely satisfied”; “mostly satisfied”; “mostly dissatisfied” and “completely dissatisfied”. The Likert scale was included in this study.

Some of the data from the self-assessment scales and the client satisfaction Likert scale were collected before a decision was made to analyse it, and consent was sought by mail from these patients. After the formal decision was taken, those patients who subsequently applied to participate in the program were given both the relevant information and the consent form by hand. The 26 patients that declined or did not reply all belonged to the group where consent was sought retrospectively. 

### 3.3. Quantitative Analysis

For each of the three instruments used, a Wilcoxon analysis was carried out using SPSS version 27. The Wilcoxon analysis was chosen, as the instruments consisted of ordinal scales, meaning the participant estimates an experience and equidistance between the options cannot be guaranteed. For each of the instruments, the results from the first questionnaire (before starting participation) were compared with the results from the second questionnaire (at the end of the program), then the third (three months after the end of the program) and then the fourth (six months after the end of the program). Comparisons were also made between the results of time 2 and times 3 and 4; in the same way, the results of time 3 were compared to time 4. Median with the statistical dispersion of quartiles, *p*-value and effect size (Cohen’s D) were calculated [93]. As it is not unusual in the field that a *t*-test analysis is performed on these kinds of instruments instead of a Wilcoxon analysis, it could be argued that it would be favourable here as well to be able to make easier comparisons. For this reason, a *t*-test was also carried out for each of the calculations. For each operation, answers from each set of questionnaires were compared, showing how many of the scores went up or down or remained the same between measurement times. Age was correlated with outcome, using Spearman’s rho [94]. Gender differences were not statistically examined in this study, as there were only 9% of the participants that identified as male and 91% that identified as female.

Considering the number of tests performed, to avoid mass significance, it was decided to sharpen the level of significance to *p* < 0.01. 

### 3.4. Qualitative Analysis

The evaluation form was designed to obtain feedback regarding the clinical work and anonymity was guaranteed. Using the Likert scale, the patients made a qualitative assessment of the NBR-program. Calculations were made of how many answers were received for each category. However, anonymity meant that we could not match specific answers with specific patients; thus, from the total number of replies (93), there were 26 forms we did not receive permission to use. Therefore, we could not analyse the results in great detail.

## 4. Results

When the self-reported data obtained from the participants before they began the program are compared to the data obtained directly after the 10-week program, statistically significant improvements regarding their overall health, their quality of life and their exhaustion symptoms are revealed. We could also see a statistically significant improvement in those same areas when comparing the data from before joining the program to that obtained three months after completion of the program. The same held true when comparing the data from before attending the program to data collected six months after finishing the program, which suggests that the improvement lasted over time. Below, we present our findings in more detail.

A significant difference in result scores was found when comparing the results of VAS from the first day of the program to the last day of the program, three months after the program and six months after the program. Comparisons of median values at different measurement times and statistical significance (*p* < 0.01) are presented in Table 1. Regarding changes between the data collected directly after the program and three months after the program, the results show no statistically significant improvement. Further, when the data obtained directly after finishing the program are compared to the data collected six months after the end of the program, the results show no statistically significant improvement. Investigating change in score over time, the categories *Improved score*, *No change in score* and *Worsened score* were used, with *Improved score* having the highest percentage for all comparisons.

A significant difference in result scores was found when comparing the results of BBQ from the first day of the program to the last day of the program, three months after the program and six months after the program. Comparisons of median values at different measurement times and statistical significance (*p* < 0.01) are presented in Table 2. Regarding change between the data collected directly after the program and three months after the program, the results show no statistically significant improvement. Further, when the data obtained directly after finishing the program are compared to the data collected six months after the end of the program, the results show no statistically significant improvement. Investigating change in score over time, the categories *Improved score*, *No change in score* and *Worsened score* were used, with *Improved score* having the highest percentage for all comparisons.

A significant difference in result scores was found when comparing the results of SMBQ from the first day of the program compared to the last day of the program, three months after the program and six months after the program. Comparisons of median values at different measurement times and statistical significance (*p* < 0.01) are presented in Table 3. When looking at change between the data collected directly after the program and three months after the program, the results show statistical improvement. Further, when the data obtained directly after finishing the program are compared to the data collected six months after the end of the program, the results show no statistically significant improvement. Investigating change in score over time, the categories *Improved score*, *No change in score* and *Worsened score* were used, with *Improved score* having the highest percentage for all comparisons. Even though a large part of the participants improved in health, we could also see that some did not experience any difference in measured health aspects, while some even experienced worse health.

The *t*-test shows the same results in terms of statistical significance as the Wilcoxon did, with the exception of the comparison of exhaustion symptoms directly after the program (SMBQ2) and six months after the program (SMBQ6), where the Wilcoxon analysis shows a statistically significant decrease in symptoms but the *t*-test analysis only shows a strong trend in the same direction. 

No significant correlations between outcome and age were found regarding overall health (VAS), as presented in Table 4.

Results obtained from the first day of the program, regarding quality of life (BBQ1) and six months after completion of the program (BBQ6), show a significant correlation between lower age and higher life-quality six months after finishing the program, as presented in Table 5. No other correlations were found.

No significant correlations between outcome and age were found regarding exhaustion symptoms (SMBQ), as presented in Table 6.

The Likert scale used to measure patient satisfaction with the program showed that all patients reported that they were either “completely satisfied” or “mostly satisfied”. As stated above, we had no way to accurately determine which answers came from which patient. Therefore, we cannot accurately state how many in each answer category were generated from the group of patients who had given their permission, but, as all of the forms reported that they were either “completely satisfied” or “mostly satisfied”, this was also true for those patients who gave permission.

## 5. Discussion

### 5.1. Results Discussion

The study shows positive results, but, even though a large percentage of the participants had improved in health, we could also see that some did not experience any difference in measured health aspects, while some even experienced worse health. There are many possible ways of considering these results; they could, for example, indicate a poor match regarding the level of illness in invited patients and intensity of the program, or even that other important mediators were influential. One factor that could influence these results is that the program partially focuses on recognising one’s symptoms and accepting the reality of the illness—which could actually indicate progress from a clinical perspective. 

Though the results show that the health of the participants improved, because of the study design, we cannot isolate the causal factors responsible for change. As previously mentioned, the setting was not controlled for strict scientific research. Therefore, unknown factors can play a role both in improvement to and decline in the measured health variables, as in the general clinical population. As we did not have a control group, we cannot know how the results would compare to no treatment or treatment as usual.

A significant correlation between outcome and age was found. This suggests that the age factor might play a role in the positive outcome of higher life-quality, but this needs to be investigated further for any conclusions to be drawn. With a larger study sample and more participants in different age groups, as well as gender groups, we could possibly obtain more correlations. As this is an actual clinical setting, it is interesting to observe what age and gender groups are referred to this kind of program. It raises questions concerning the onset of stress-related illness, as well as whom is identified as a candidate for the program in the healthcare system. 

The results from the evaluation form shows that all participants were completely satisfied, or mostly satisfied, with participation in the program. Due to the study design, we cannot present how many were in each category, which would have been desirable.

Although Exhaustion Syndrome can vary in severity, rehabilitation often takes a relative long time. The results in this study measure change after participation in a 10-week program where patients take part 2 times per week for 3 h. Previous studies show that three of the documented and better-known programs in Sweden included the following variations: 28 weeks (16 weeks with the possibility of 12 more weeks) at 4 half-days per week [53]; 14 weeks, 3 times per week for 3.5 h [40] and 28 weeks, starting the first week with 1 day of attendance and then increasing the time over 4 weeks until 4 half-days per week is reached [31]. The studied NBR-program is fairly short in comparison, and puts the positive results in an interesting light [31,40,53]. In summary the outcomes of this study are in line with previous research and suggest that the medical NBR-program shows promising results, although further research is needed. 

### 5.2. Methodological Discussion

The study was carried out as an examination of an established real-world clinical setting, a naturalistic field study, as opposed to a program designed for research. From a pure methodological perspective, this involves certain limitations in comparison to the “gold standard” for research, as the study had no control group or randomisation. Also, there was a broad criterion for patient group selection in the already existing program, which, by extension, was also true for the participants in the present study. On the other hand, it is of interest for, amongst others, caregivers, consumers, politicians and taxpayers to look at clinical programs that are established parts of the existing healthcare system. The idea to study the program was introduced by one of the team members after six patient groups had participated, which can be seen as a possible source of bias, even though that team member then stopped working with the program to carry out the research. The instruments were chosen to examine the effectiveness of the clinical setting, with no future study in mind. Afterwards, it is obvious that, for research purposes, it would have been interesting to collect more data. When it comes to the qualitative method, limitations occurred when using the data for research purposes rather than clinical purposes as the material was collected anonymously and, therefore, could not be very precisely presented. It would have been desirable to be able to present the number of answers in each Likert category rather than presenting them in broader terms; if it would have been designed for study purposes to begin with, this could have been solved. The three instruments used in this study all have satisfying validity and reliability. The reliability for BBQ is on the lower side, but, as it is considered to be as good as its widely used predecessor QOLI, we felt comfortable including it in the study.

It would have been interesting to include more qualitative results from the questionnaire in the study and/or to complement them with interviews. This would have given a deeper insight into the participants’ experience and might have shed some light on perceived mediators. For example, the participants reported that it was of benefit to meet people in similar situations to their own, to participate in a structured activity while on sick-leave and to learn how they themselves could incorporate elements of nature into their everyday life to continue to improve their own health.

## 6. Conclusions

This research shows that participation in the program led to a higher quality of life, lesser exhaustion symptoms and improved overall health. The results were still statistically significant three and six months after finishing the program. From a public health perspective, and with background knowledge of the nature of the patient group under treatment, the studied program would appear to be both economical and effective. It has both a satisfied patient group and favourable comparison with the outcomes of other research programs. Nature-based programs are, in a modern sense, in their infancy, but the kinds of results shown in this study are promising and would seem to justify further investigation. The three major areas suggested for future research are the need for randomised controlled studies to establish if this kind of program is superior to no treatment and/or superior to or as good as treatment as usual treatment; investigation of what variables contribute to the positive change; longitudinal studies to investigate if the positive outcomes stand the test of time beyond six months after finishing such a program. It would be beneficial to, in addition to self-assessment scales and interviews, measure brain activity and cortisol levels. 

## Figures and Tables

**Table 1:** Differences between results obtained from VAS on the first day of the program (VAS1), the last day of the program (VAS2), three months after finishing the program (VAS3) and six months after finishing the program (VAS6). Also presented are median, effect size and difference in score of VAS over time.

	Median (P25–P75)	*p*	Cohen’s D	Improved Score N (%)	No Change in Score N (%)	Worsened Score N (%)
VAS1	40 (30–50.25)	0.001 **	−0.447	40 (60.6)	8 (12.1)	18 (27.2)
VAS2	50 (33.75–60)					
N = 66						
VAS1	40 (30–54)	0.001 **	−0.611	45 (75)	5 (8.3)	10 (16.6)
VAS3	55 (40–70)					
N = 60						
VAS1	50 (35–59.25)	0.004 **	−0.539	30 (71,4)	6 (14.2)	6 (14.2)
VAS6	55 (48.75–67.75)					
N = 42						
VAS2	50 (35–60)	0.018	−0.315	37 (61.7)	5 (8.3)	18 (30)
VAS3	55 (40–70)					
N = 60						
VAS2	52.5 (30–61.75)	0.064	−0.264	24 (57.1)	4 (9.5)	14 (33.3)
VAS6	60 (48.75–67.75)					
N = 42						
VAS3	58.58 (40–70)	0.631	−0.023	22 (52.3)	5 (11.9)	15 (35.7)
VAS6	60 (48.75–67.75)					
N = 42						

** *p* < 0.01.

**Table 2:** Differences between results obtained from BBQ on the first day of the program (BBQ1), the last day of the program (BBQ2), three months after finishing the program (BBQ3) and six months after finishing the program (BBQ6). Also presented are median, effect size and difference in score of BBQ over time.

	Median (P25–P75)	*p*	Cohen’s D	Improved Score N (%)	No Change in Score N (%)	Worsened Score N (%)
BBQ1	44 (31–51.75)	0.001 **	−0.484	47 (71.2)	1 (1.5)	18 (27.3)
BBQ2	48 (38–58.25)					
N = 66						
BBQ1	44.5 (34.0–53-25)	0.001 **	−0.556	42 (70.0)	1 (1.7)	17 (28.3)
BBQ3	51 (42.25–61.50)					
N = 60						
BBQ 1	46 (34–55.5)	0.004 **	−0.405	33 (75.0)	1 (2.3)	10 (22.7)
BBQ 6	52 (44–67.25)					
N = 44						
BBQ 2	48 (−39.25–58.75)	0.349	−0.147	32 (53.3)	1 (1.7)	27 (45.0)
BBQ3	51 (42.25–61.50)					
N = 60						
BBQ 2	48 (38.50–56)	0.163	0.199	25 (56.8)	1 (2.3)	18 (40.9)
BBQ 6	52 (44–67.25)					
N = 44						
BBQ 3	54 (40.75–63)	0.649	0.001	24 (54.5)	0 (0)	20 (45.4)
BBQ 6	52 (43–67.25)					
N = 44						

** *p* < 0.01.

**Table 3:** Differences between results obtained from SMBQ on the first day of the program (SMBQ1), the last day of the program (SMBQ2), three months after finishing the program (SMBQ3) and six months after finishing the program (SMBQ6). Also presented are median, effect size and difference in score.

	Median (P25–P75)	*p*	Cohen’s D	Improved Score N (%)	No Change in Score N (%)	Worsened Score N (%)
SMBQ1	5.18 (4.45–5.77)	0.002 **	0.355	43 (64.2)	1 (1.5)	23 (34.3)
SMBQ32	4.91 (4.32–5.41)					
N = 67						
SMBQ1	5.14 (4.45–5.57)	0.001 **	0.544	39 (63.9)	1 (1.6)	21 (34.4)
SMBQ3	4.59 (3.67–5.38)					
N = 61						
SMBQ1	5.09 (4.18–5.54)	0.001 **	0.582	33 (76.7)	0 (0)	10 (23.2)
SMBQ6	4.59 (3.59–5.18)					
N = 43						
SMBQ2	4.91 (4.32–5.38)	0.019 **	0.365	38 (62.3)	1 (1.6)	22 (36.1)
SMBQ3	4.59 (3.67–5.38)					
N = 61						
SMBQ2	4.73 (4.22–5.18)	0.019 **	0.301	25 (58.1)	1 (2.3)	17 (39.5)
SMBQ6	4.59 (3.59–5.18)					
N = 43						
SMBQ3	4.55 (3.41–5.50)	0.121	0.086	26 (60.5)	0 (0)	17 (39.5)
SMBQ6	4.59 (3.59–5.18)					
N = 43						

** *p* < 0.01.

**Table 4:** Correlation between outcomes of VAS and age, with correlation coefficient (Rs), *p* value and confidence interval.

	N	Spearman’s Rho	CI * 95%	Sig. *p*
Diff. VAS1/VAS2 and age	66	0.159	(−0.093–0.393)	0.201
Diff. VAS1/VAS3 and age	60	0.002	(−0.259–0.263)	0.988
Diff. VAS1/VAS6 and age	42	0.046	(−0.271–0.353)	0.775

* Confidence interval.

**Table 5:** Correlation between outcomes of BBQ and age, with correlation coefficient (Rs), *p* value and confidence interval.

	N	Spearman’s Rho	CI * 95%	Sig. *p*
Diff. BBQ1/BBQ2 and age	66	0.008	(−0.256–0.241)	0.949
Diff. BBQ1/BBQ3 and age	60	0.004	(−0.257–0.256)	0.973
Diff. BBQ1/BBQ6 and age	44	−0.464	(−0.673–−0.185)	0.002

* Confidence interval.

**Table 6:** Correlation between outcomes of SMBQ and age, with correlation coefficient (Rs), *p* value and confidence interval.

	N	Spearman’s Rho	CI * 95%	Sig. *p*
Diff. SMBQ1/SMBQ2 and age	67	−0.120	(−0.356–0.131)	0.335
Diff. SMBQ1/SMBQ3 and age	61	−0.108	(−0.357–0.155)	0.408
Diff. SMBQ1/SMBQ6 and age	43	0.087	(−0.386–0.227)	0.577

* Confidence interval.

## Data Availability

The collected data are stored in a safe at the studied establishment.

## Appendix A

**Table A1:** Description of themes and main activities.

Week	Day 1	Day 2
1	Introduction to the program, team members and facilities. Walk in nature.	Short lecture about stress. Activity with guinea pigs. Walk in nature.
2	Introduction to CBT and ACT. Walk in nature.	Walk in nature, including wood-carving activity
3	Activity balance and life balance. Walk in nature.	Activity with calves. Walk in nature.
4	The brain and stress: getting to know your signals. Walk in nature	Perennial sowing. Walk in nature.
5	Behaviour: behavioural analysis. Walk in nature.	Wood carving and taking currant cuttings. Walk in nature.
6	Acceptance. Walk in nature.	Making Christmas decoration out of natural materials. Walk in nature.
7	Thoughts: recognise and manage thoughts. Walk in nature.	Dry-felting. Walk in nature.
8	Life compass: living according to your values. Walk in nature.	Meeting horses. Walk in nature.
9	Going back to work: maintenance. Walk in nature.	Individual check-in with patients. Choice between gardening and dry-felting.
10	Closing session for the psychological group treatment, summary and maintenance. Walk in nature.	Closing session for the program. Walk in nature, including grilling over open fire, completion of scales and evaluation and summary of the program.
